# Application of a New Type of Protective Sputum Suction Device in Clinic against Cross-Infection between Medical Staff and Patients

**DOI:** 10.1155/2023/9927819

**Published:** 2023-12-31

**Authors:** Kang Lu, Weizhou Wu, Gaomei Jin, Haixia Yang, Xiaowei Cai, Lin Li, Zanchao Chen, Lin Ren, Baoshuan Guo, Qing-feng Xue

**Affiliations:** ^1^Cadre Ward Department, The 985th Hospital of the Joint Logistics Support Force, Taiyuan, China; ^2^Neurology Department, The 985th Hospital of the Joint Logistics Support Force, Taiyuan, China; ^3^Dermatology Department, The 985th Hospital of the Joint Logistics Support Force, Taiyuan, China; ^4^Clinical Laboratory, The 985th Hospital of the Joint Logistics Support Force, Taiyuan, China; ^5^Disease Prevention Department, the 985th Hospital of the Joint Logistics Support Force, Taiyuan, China; ^6^Hematology and Oncology Department, The 985th Hospital of the Joint Logistics Support Force, Taiyuan, China; ^7^Henan Di Yi Medical Technology Development Co., Ltd., Changyuan Country, Xinxiang City, Henan 453400, China; ^8^Anesthesiology Department, The 985th Hospital of the Joint Logistics Support Force, Qiaodong Road No. 30, Taiyuan 030001, China

## Abstract

**Objective:**

To explore the clinical application of a new type of protective sputum suction device (PSSD) in patients with tracheotomy or tracheal intubation and to evaluate the protective effect of PSSD against cross-infection between medical staffs and patients.

**Methods:**

A novel PSSD was designed which can assist closed sputum suction operation without disconnecting the ventilator. 32 patients with tracheotomy were included to study the protective effect and safety of this device. Patients' vital signs including heart rate, respiratory rate, mean arterial pressure, and blood oxygen saturation were recorded to compare the influence of open suction and closed suction (performed with this novel device). To verify the antisplash effect of this device on airway secretions, bacterial samples were collected from the hands of the suction operators and the environment near the endotracheal tube orifice before and after the two suction processes. In addition, the satisfaction of the two suction methods was compared through the questionnaire of suction staff. Finally, with the assistance of this device, an attempt was made to complete the bronchoscopy without weaning of ventilator.

**Results:**

Compared with open sputum suction, closed sputum suction has a smaller decrease in patients' blood oxygen saturation (*P* < 0.05), and no significant differences in other vital signs. Compared with open sputum suction, bacteria from the hands of suction staffs and the surrounding environment of the endotracheal tube were barely detected in closed suction. A questionnaire survey of sputum suction nurses suggested that the satisfaction with use and protective effect of the closed suction were better than open suction. In addition, bronchoscopy can be successfully completed with the assistance of this device, which is not possible for other breathing tubes.

**Conclusion:**

This closed sputum suction device has little effect on the oxygen saturation of patients but has excellent protective effects for medical staff against cross-infection. It has a unique advantage that can assist in completing the fiberoptic bronchoscopy with continuous ventilator-assisted breathing.

## 1. Introduction

Trachea intubation [1] and tracheotomy [2, 3] are common operations in the intensive care unit (ICU), emergency department, and operating room to provide respiratory support to patients through a ventilator. Up to 20 million patients worldwide are mechanically ventilated each year [4]. Under mechanical ventilation, the patient's ability to spontaneously expectorate is weakened. So, sputum suction becomes an important task of airway management for patients with tracheal intubation or tracheotomy [5, 6].

In the past, the most commonly used suction technique was “open suction (OS),” which needs disconnecting the ventilator and then suctioning the patient's airway. However, disconnecting the breathing opportunity resulted in a significant drop in airway pressure, reduced lung volume, and decreased oxygen saturation. Another important point is that the aerosols from patients with respiratory infectious diseases easily infect medical staffs, especially during open sputum suction [7]. Therefore, various closed suction (CS) methods have been widely developed. Some studies showed that CS has more advantages than OS in stabilizing blood oxygen saturation and reducing ventilator-associated pneumonia [8].

However, some completely closed suction tubes have some unavoidable problems, such as incomplete withdrawal of the suction catheter due to catheter sheath twisting [9]. In addition, for patients with endotracheal intubation or tracheotomy, fibroptic bronchoscopy is sometimes inevitable. This operation would not be performed if the patient needed continuous respiratory support because there is no extra channel to place a bronchoscope. In addition, the completely closed suction tube with an outer sheath will lead to sputum residue after repeated use, breeding more bacteria and blocking the suction port, making it difficult to enter and leave the suction port smoothly.

To solve these problems, we designed a new type of PSSD, which makes sputum suction easier and more protective for nurses. Meanwhile, with this device, patients can get uninterrupted ventilator support, thus avoiding the risk of weaning from the ventilator during suction.

## 2. Protective Sputum Suction System Design

### 2.1. Design Concept

**Figure 1:**
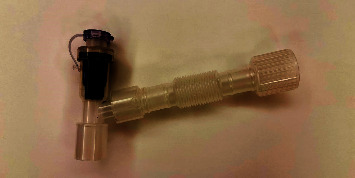
Picture of protective sputum suction device product.

The system was designed to be connected between the endotracheal catheter and ventilator circuit. This device can assist in sputum suction operation without disconnecting the ventilator. It has the function of a one-way valve means the suction channel allows the suction tube to pass smoothly and keep it continuously closed, while the air in the respiratory tract cannot be discharged to the surroundings. When the suction tube is pulled out, the suction channel is sealed by itself. It will mostly reduce the risk of infection for medical staff during the sputum suction process. This device has obtained independent intellectual property rights protection (China National Invention Patent No. ZL 2019 1 0141806.5) (Figure 1).

### 2.2. Components

#### 2.2.1. Outer Shell

**Figure 2:**
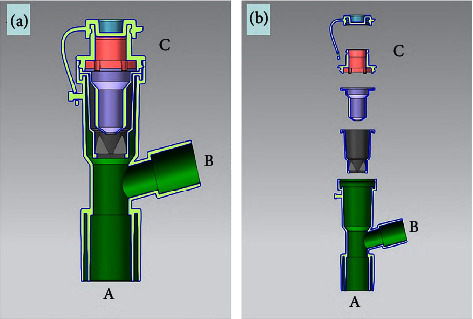
Physical diagram of protective sputum suction device. Connection port *A* is used to connect the tracheostomy catheter or endotracheal tube. Connection port *B* is used to connect the breathing circuit of the ventilator. Operation channel *C* is for sputum suction, which is closed after using.

It was designed as a T-shaped three-way connecting pipe with two standard connectors (Figure 2). Connection port *A* is used to connect the tracheostomy catheter or endotracheal tube. Connection port *B* is used to connect the breathing circuit of the ventilator. If the patient does not require mechanical ventilation, a T-piece using an HME with expiratory filter may be connected to the connection port *B* to maintain a closed system. The remaining nozzle (Figure 2(C)) is for sputum suction.

#### 2.2.2. Closed Sputum Suction System

**Figure 3:**
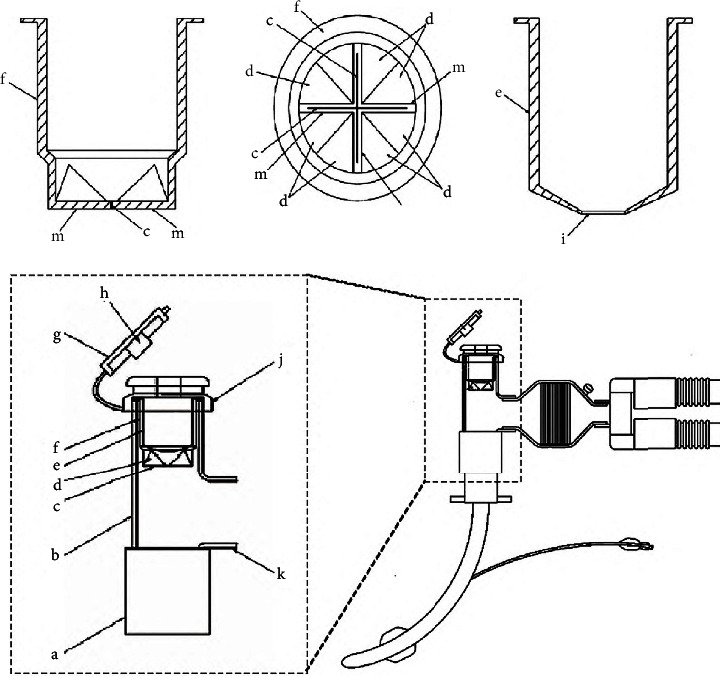
Detail structure of protective sputum suction device. (a) Lower nozzle of adapter, (b) adapter, (c) rib, (d) pressure bevel, (e) stop sleeve, (f) isolation sleeve, (g) sealing cover, (h) plug body, (i) circular hole, (j) sealing ring, (k) side nozzle of adapter, and (m) groove seam.

It consists of a cross-split seal sleeve and a stop sleeve, both of which are made of soft rubber (Figure 3). The stop sleeve is embedded in the cross-split seal sleeve, and the two are tightly bonded to the outer shell together by sealing ring (Figure 3(j)). There are four splits (Figure 3(c)) and eight incline planes (Figure 3(d)) at the bottom of seal sleeve (Figure 3(f)). When the suction tube is inserted, the cruciform structure will be propped open, and when the suction tube is withdrawn, it will be closed by the positive pressure in the trachea. The air tightness of the splits increases as the endotracheal pressure on the incline plane increases. There is a small circular hole (Figure 3(i)) at the bottom of the stop sleeve (Figure 3(e)), which can allow the suction tube to pass through. There is an interference fit between the hole and the suction tube, so the airway secretion would not spray out. The function of stop sleeve is not only to increase the sealing performance but also to prevent the cruciform structure from rolling out when the suction tube is withdrawn.

#### 2.2.3. Heat and Moisture Exchanger (HME)

HME is detachably connected with connection port *B*. If the patient does not require mechanical ventilation, HME with expiratory filter will maintain a closed system. HME can collect and retain the heat and water in the exhaled air. When inhaling, the air passes through the HME and is brought into the airway in a humid and warm state. In addition, there is a carbon dioxide monitoring port next to the HME to monitor carbon dioxide concentration.

## 3. Methods

### 3.1. Patient Population and Ethical Review

A total of 32 consecutive patients with tracheotomy in intensive care unit were included in the study. This study is approved by the Ethics Committee of 985th hospital. All programs executed in related research meet morals. Informed consent was obtained from all individual patients included in this study. All of the medical records were anonymous, and no patient information was extracted except for research purposes.

### 3.2. Suctioning Procedures

In this study, each patient received two episodes of suctioning, open suction (OS) and then closed suction (CS). The symptoms needed for suctioning, which were diagnosed by the medical staffs in the ICU ward, were excessive secretions, abnormal lung sounds, and excepted decreased oxygen saturation (SaO_2_) to prevent experimental errors. Suctioning was performed by the nurse when it was required.

To eliminate the effects of these two suction methods, we spaced at least 24 hours between the two suctioning methods to ensure that the patient returned to the same baseline level of vital signs. The suctioning techniques were done with respect to the acceptable standards [10]. The suctioning time in each episode was around 15 seconds. Before the open suction operation, we began with hyperoxygenating the patients and disconnecting them from ventilator. In the suction process, safety equipment items such as sterile gloves, protective glasses, and face mask were necessary to prevent contamination with patient's respiratory secretions.

### 3.3. Detection of Vital Signs

Compared with open sputum suction, the most obvious advantage of closed sputum suction for patients is that continuous ventilator support breathing can be carried out during sputum suction, which can avoid a series of fluctuations of vital signs caused by transient hypoxia. Especially for severe patients, fluctuations in hemodynamics and blood oxygen saturation caused by repeated weaning from the ventilator may cause severe consequences. We recorded the vital signs of the same patient before and after both of OS and CS, including heart rate, respiratory rate, mean arterial pressure, and blood oxygen saturation. All of these data were analyzed to demonstrate the superior effect of the PSSD on the general vital signs of patients (all data collection was repeated three times).

### 3.4. Protective Effect of PSSD on the Prevention of Bacterial Cross-Infection in Medical Staff

In open suction (OS), the operator needs to disconnect the ventilator and endotracheal intubation. A large number of gas coercing secretions will splash into the surrounding environment through the airway and respiratory circuit in a moment. These airway secretions will cause potential infection hazards to medical staff. Oppositely, the closed sputum suction avoids the disconnect ventilator, so that there is no airway secretions splintered outward during the whole operation. Bacteria from the hands of suction nurses and the surrounding environment within the splitter range of the endotracheal tube were sampled and cultured to evaluate the protective effect of PSSD to medical workers and the environment (the experiment was repeated three times). Before bacterial sampling, the operator wore sterile gloves and spread sterile sheets around the endotracheal tube. After sputum suction, samples were taken from the operator's hand where they might be sprayed, and samples were taken within 30 cm of the endotracheal tube.

### 3.5. Nurses' Satisfaction with the Closed Suction Method by PSSD

A questionnaire survey was conducted among 25 nurses participating in open and closed sputum suction operations. The two methods of sputum suction were scored anonymously by hundred-mark system including the convenience of sputum suction operation (10 points, a score of 0 represents the least convenient and a score of 10 represents the most convenient), satisfaction with protective effect (10 points, a score of 0 represents the worst protection and a score of 10 represents the best protection), and subjective evaluation of sputum suction effect (10 points, a score of 0 represents the least effective suction and a score of 10 represents the best). The three ratings were summed and converted to a hundred score, which was used as the final satisfaction score for statistical analysis.

### 3.6. To Verify the Feasibility of Continuous Operation of Fiberoptic Bronchoscopy through the PSSD

In clinic, many patients with tracheal intubation need to undergo fiberoptic bronchoscopy or suction from the deep of lung, which usually requires disconnecting the ventilator circuit. However, patients who need continuous mechanical ventilation cannot tolerate prolonged deoxygenation for fiberoptic bronchoscopy. An additional functional advantage of the PSSD is the feasibility of performing fiberoptic bronchoscopy while the ventilator is continuously turned on. To detect its safety, we performed fiberoptic bronchoscopy though the PSSD and recorded the oxygen saturation of patients per 5 minutes throughout the procedure.

## 4. Data Analysis

All data were analyzed by using SPSS 18.0 software. Paired sample *T* test analysis was used to analyze the differences vital signs before and after OS and CS. Independent sample *T* test was used to test the subjective rating of the two suction methods. *P* < 0.05 was considered as statistically significant.

## 5. Results

### 5.1. Patients' Characteristics

From 32 patients who participated in the study, 84.4% (27 patients) were male. The age of patients was between 38 and 92 years (74.5 ± 12.8, by mean) (Table 1).

### 5.2. The Closed Suction Method Has Less Adverse Effect on the Vital Signs

The mean heart rate before and after OS was 74.3 ± 12.4 and 102.8 ± 12.2, before and after CS was 77.3 ± 12.9 and 103.3 ± 10.5. The average respiratory rate of patients before and after OS was 19.8 ± 4.6 and 27.6 ± 5.2, before and after CS was 20.7 ± 5.4 and 25.8 ± 5.2. The mean arterial pressure of the patients was 93.7 ± 15.9 and 98.5 ± 15.8 before and after OS, before and after CS was 97.2 ± 14.7 and 92.6 ± 14.8. There were no significant differences between the OS and CS method in value of variation (*p* < 0.05), although the mean fluctuation of heart rate, respiratory rate, and mean arterial pressure during closed suction was smaller than that during open suction. Oxygen saturation was 95.8 ± 2.4% before open suction, 91.0 ± 3.8% after open suction, 95.2 ± 3.3% before closed suction, and 96.4 ± 2.5% after closed suction. The oxygen saturation of patients after OS was significantly lower than that in CS (*P* < 0.05) (Table 2).

### 5.3. The PSSD Has Significantly Better Bacterial Protection Effect on Medical Staff

**Figure 4:**
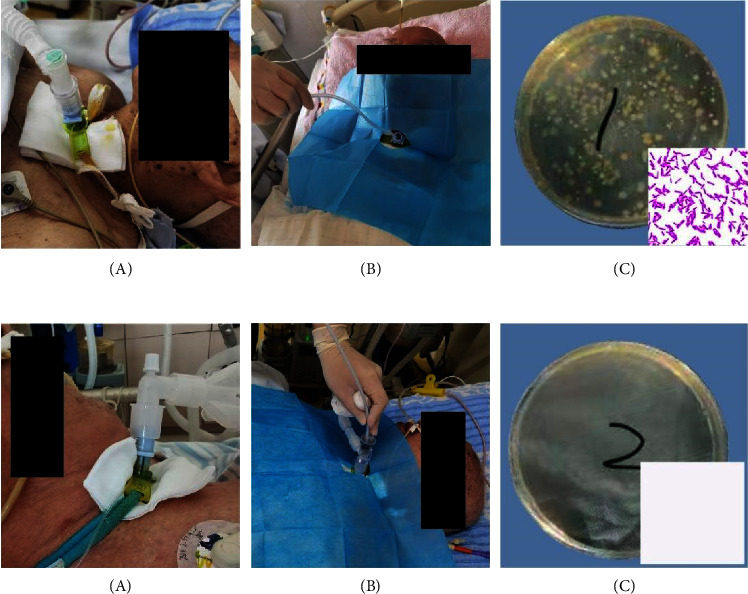
Sampling and comparison of bacteria on surrounding objects under OS and CS sputum aspiration. (a) A: common breathing circuit without PSSD; B: open sputum suction in patients with tracheotomy; C: acinetobacter baumanii was detected in hand and environmental bacterial sampling after open sputum suction. (b) A: breathing circuit with PSSD; B: closed sputum suction in patients with tracheotomy; C: no bacterial growth was detected in hand and environmental bacterial sampling after closed sputum suction.

No bacteria were cultured from the aspirator's hands or from the surrounding environment both before OS and CS methods. The average positive rate of hand bacteria sampling culture was 71.9%, and the average environment bacteria sampling culture was 36.5% in 32 cases after OS. No bacterial growth was detected in hand and environmental bacterial sampling after CS (Figure 4).

### 5.4. Nurses Are More Satisfied with the PSSD

The convenience, protective effect, and suction effect of the two suction methods were scored by the sputum suction staff. In terms of convenience, the score of PSSD-assisted suction method and OS suction method was 6.56 ± 1.15 and 5.40 ± 1.44, respectively (*p*=0.03). The scores of protection effect of PSSD and OS method were 7.88 ± 1.09 and 2.48 ± 1.16, respectively (*p*=0.001). The scores of suction effect of PSSD and OS method were 8.12 ± 0.97 and 8.08 ± 1.35, respectively (*p*=0.905). There was a significant difference between the two methods in terms of convenience and protection effect, while there was no significant difference in suction effect (Table 3).

### 5.5. Fiberoptic Bronchoscopy Can Be Performed with the Aid of the PSSD

**Figure 5:**
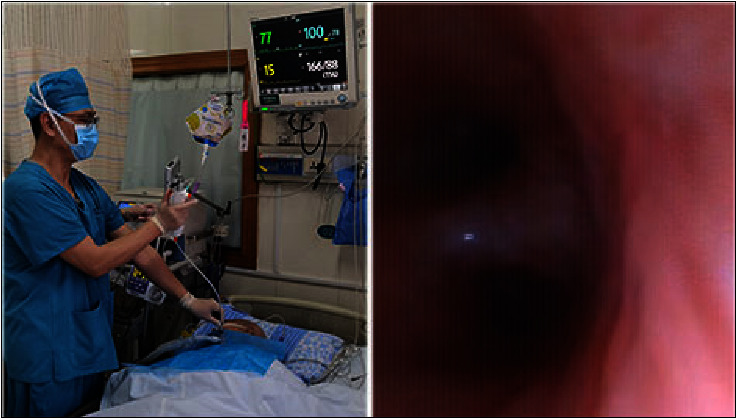
Bronchoscopic operation assisted by protective sputum suction device product. Under the monitoring of vital signs, bronchoscopy and endoscopic sputum aspiration were performed through the breathing pipe with PSSD, and the patient's vital signs almost did not fluctuate owing to the continuous work of the ventilator.

Fiberoptic bronchoscopy was performed in 10 patients who needed deep sputum aspiration. The main indications were massive phlegm, wet rales in the lungs, and partial atelectasis. Through the PSSD, we performed deep sputum suction using fiberoptic bronchoscopy with continuous ventilator support, and the whole operation process was successfully performed. No decrease in blood oxygen saturation and obvious fluctuations of vital signs during the operation was observed. However, the respiratory circuit of open sputum suction did not have fiberoptic bronchoscopy operation channel. If the ventilator is disconnected, the patient's oxygen saturation decreases rapidly. No patient can tolerate deep suction operation in a state of prolonged hypoxia (Figure 5).

## 6. Discussion and Conclusion

The closed suction system is increasingly used because of its advantages compared to the conventional, open suction system (OSS), including less time involved, better patient tolerance because of fewer physiologic disturbances [11]. Previous studies have shown that closed suction systems may better prevent late-onset ventilator-associated pneumonia (VAP) [12, 13]. Moreover, it can be helpful in limiting environmental, personnel, and patient contamination and in preventing the loss of lung volume and the alveolar derecruitment associated with standard suctioning in the severely hypoxemic patients [14]. Performing suctioning without disconnecting the patient from the ventilator has also been recommended for adults with high FiO_2_, or PEEP, or at risk for lung derecruitment, and for neonates by AARC clinical practice guidelines [15].

Despite the benefits, there are some inevitable problems with the closed sputum suction system widely used now. For the closed suction tube with an external sheath, after a number of suctioning procedures, the soft catheter sheath may twist, leading to the shortening of its length, making the sputum aspiration tube unable to withdraw in time and thus increasing airway resistance [9]. Similarly, Bhattacharjee's study found that during sputum aspiration care for COVID-19 patients, incompletely withdrawn of the closed suction catheter and the rotating access knob not be closed may occurred. Twist catheter sheath and impaired visibility due to fogging of goggles may lead to inadvertent errors while operating closed suction catheter system [16].

In this paper, we design a close sputum suction device for patients with trachea intubation or tracheotomy. Its main functions and advantages are as follows: (1) prevent respiratory droplets or aerosols from splashing into the air during sputum aspiration, so as to reduce the risk of infection of medical personnel; (2) there is no need to disconnect the continuous ventilator to assist breathing during sputum aspiration, so as to maintain the oxygen supply of patients; (3) fiberoptic bronchoscopy is feasible during continuous mechanical ventilation; (4) prevent foreign bodies or pathogenic bacteria in the air from entering the lungs of patients in the tracheotomy status; (5) it has a wide range of application and is more convenient and cheap.

The ingenious design of this new type of closed suction device is the construction of cross fissure, and stop sleeve make the airway completely closed when the sputum is aspirated or not. Therefore, during the whole process of sputum suction or after the suction tube is removed, the secretions in the patient's airway will not spill out and contaminate the surrounding environment and medical staff. By sampling the bacteria in the environment and the hands of sputum aspirators under open and closed suction methods, we found that the PSSD could prevent respiratory tract bacteria from contaminating the surrounding environment and greatly avoid cross-infection of patients to medical staff.

In our study, we found that this closed suction method has more advantages than the open suction to disturb the vital signs of patients, especially the fluctuation of blood oxygen saturation. Even some patients, blood oxygen saturation did not return to the original baseline level after the open suction seven minutes later. We have reason to doubt that, the short-term decrease in oxygen saturation caused by open suction can lead to hypoxia of vital organs. Further studies are needed to confirm this point.

## 7. Limitations and Prospect

Although there are many advantages, the PSSD with no casing may bring up other issues compared with fully airtight suction tube with sheath. The suction tube will still be exposed to the air at the end of the suction. So, for patients with infectious diseases, the suction operator must wear gloves and operate carefully, and disinfect their hands in time to avoid the contamination of the surrounding environment by the suction tube.

It is worth looking forward to that through the application of this device, the cross infection between doctors and patients can be greatly reduced, and the difficulty of closed suctioning operation will be further reduced. Meanwhile, the psychological burden of nurses serving patients with tracheotomy can be significantly reduced. For general patients, especially in underdeveloped areas, this device is more economical and worthy of clinical promotion. More clinical observations and further studies will be needed to improve this device.

## Figures and Tables

**Table 1:** Demographic characteristics of including patients.

Total	Gender		Age		
	Male	Female	30–49	50–69	>70
32	27	5	2	7	23

**Table 2:** Comparison of vital signs before and after OS and CS.

	Technique	Before suction	After suction	*P* value
Heart rate	OS	74.3 ± 12.4	102.8 ± 12.2^*∗*^	0.001
	CS	77.3 ± 12.9	103.3 ± 10.5^*∗*^	0.001
	*P* value	0.269	0.828	

Respiration rate	OS	19.8 ± 4.6	27.6 ± 5.2^*∗*^	0.001
	CS	20.7 ± 5.4	25.8 ± 5.2^*∗*^	0.001
	*P* value	0.472	0.162	

Mean arterial pressure	OS	93.7 ± 15.9	98.5 ± 15.8	0.273
	CS	97.2 ± 14.7	92.6 ± 14.8	0.241
	*P* value	0.416	0.166	

Oxygen saturation (SaO_2_)	OS	95.8 ± 2.4	91.0 ± 3.8^*∗*^	0.001
	CS	95.2 ± 3.3	96.4 ± 2.5	0.149
	*P* value	0.320	0.001^*∗*^	

^*∗*^: there is a significant difference compared with before suction group; CS, closed suction; OS, open suction.

**Table 3:** Subjective rating of PSSD-assisted suction (CS) and OS by sputum operators.

	Convenience of use	Effect of protection	Effect of suction
CS	6.56 ± 1.15	7.88 ± 1.09	8.12 ± 0.97
OS	5.40 ± 1.44^*∗*^	2.48 ± 1.16^*∗*^	8.08 ± 1.35^*∗*^
*p*	0.003	0.001	0.905

^*∗*^: there is a significant difference compared with CS group; CS, closed suction; OS, open suction.

## Data Availability

The data used to support the findings of this study are included within the article.
